# Winter roost selection of Lasiurine tree bats in a pyric landscape

**DOI:** 10.1371/journal.pone.0245695

**Published:** 2021-02-09

**Authors:** Marcelo H. Jorge, W. Mark Ford, Sara E. Sweeten, Samuel R. Freeze, Michael C. True, Michael J. St. Germain, Hila Taylor, Katherine M. Gorman, Elina P. Garrison, Michael J. Cherry

**Affiliations:** 1 Department of Fish and Wildlife Conservation, College of Natural Resources and Environment, Virginia Polytechnic Institute and State University, Blacksburg, VA, United States of America; 2 U.S. Geological Survey, Virginia Cooperative Fish and Wildlife Research Unit, Blacksburg, VA, United States of America; 3 Conservation Management Institute at Virginia Polytechnic Institute and State University, Blacksburg, VA, United States of America; 4 Caesar Kleberg Wildlife Research Institute, Texas A&M University-Kingsville, Kingsville, TX, United States of America; 5 Florida Fish and Wildlife Conservation Commission, Gainesville, FL, United States of America; Bowling Green State University, UNITED STATES

## Abstract

Day-roost selection by Lasiurine tree bats during winter and their response to dormant season fires is unknown in the southeastern United States where dormant season burning is widely applied. Although fires historically were predominantly growing season, they now occur in the dormant season in this part of the Coastal Plain to support a myriad of stewardship activities, including habitat management for game species. To examine the response of bats to landscape condition and the application of prescribed fire, in the winter of 2019, we mist-netted and affixed radio-transmitters to 16 Lasiurine bats, primarily Seminole bats (*Lasiurus seminolus*) at Camp Blanding Joint Training Center in northern Florida. We then located day-roost sites to describe roost attributes. For five Seminole bats, one eastern red bat (*Lasiurus borealis*), and one hoary bat (*Lasiurus cinereus*), we applied prescribed burns in the roost area to observe bat response in real-time. Generally, Seminole bats selected day-roosts in mesic forest stands with high mean fire return intervals. At the roost tree scale, Seminole day-roosts tended to be larger, taller and in higher canopy dominance classes than surrounding trees. Seminole bats roosted in longleaf (*Pinus palustris)*, slash (*Pinus elliotii*) and loblolly pine (*Pinus taeda*) more than expected based on availability, whereas sweetbay (*Magnolia virginiana*), water oak (*Quercus nigra*) and turkey oak (*Quercus laevis*), were roosted in less than expected based on availability. Of the seven roosts subjected to prescribed burns, only one male Seminole bat and one male eastern red bat evacuated during or immediately following burning. In both cases, these bats had day-roosted at heights lower than the majority of other day-roosts observed during our study. Our results suggest Seminole bats choose winter day-roosts that both maximize solar exposure and minimize risks associated with fire. Nonetheless, because selected day-roosts largely were fire-dependent or tolerant tree species, application of fire does need to periodically occur to promote recruitment and retention of suitable roost sites.

## Introduction

Prescribed fire is used to restore historical disturbance regimes, alter vegetation structure, reduce fuel loads, and maintain wildlife habitat [1]. In the southeastern United States, the longleaf pine (*Pinus palustris*) ecosystem is an archetypal, fire-mediated, ecosystem with one of the shortest fire return intervals of any system in North America [1,2]. This ecosystem is characterized by structural attributes that facilitate frequent fires including fine-fuel inputs, such as pine needles with high resin content, along with wire-grass (*Aristida* spp.) that provide micro-elevation for fuel desiccation and well-ventilated fires [3,4]. Conversion to plantation forestry and agriculture or development has reduced the longleaf pine ecosystem to < 5 percent of its historical range [5,6]. Moreover, widespread fire suppression has led to forest mesophication whereby shade-tolerant and fire-intolerant tree species replace shade-intolerant and fire-tolerant tree species [7]. This often invokes a feedback loop that continually promotes hardwood invasion into a formerly pine-dominated system and further changes subsequent fire behavior when applied [8]. Consequently, two-thirds of all species of flora and fauna that are threatened, endangered, or in decline in the southeastern United States are associated with the longleaf pine ecosystem, making restoration a high priority for conservation [9].

Longleaf pine ecosystem maintenance and restoration efforts include returning prescribed fire to the landscape at regular, frequent intervals [10,11] and mechanical [12,13], as well as, chemical [14] removal of hardwoods within pine stands. In the Coastal Plain, longleaf pine communities naturally burned during the growing season [15,16]. However, many land managers utilize dormant season burning for decades because these fires burn at lower intensities, help promote northern bobwhite (*Colinus virginianu*s) habitat, do not disrupt spring or summer ground nesting herpetofauna and avifauna, and minimize risk to endangered red-cockaded woodpecker (*Leuconotopicus borealis*) cavity trees that are highly flammable [17–22].

Currently, bats are a taxa of high conservation concern in North America due to ongoing impacts of white-nose syndrome on hibernating species [23] and wind-energy impacts to migratory, non-hibernating species [24] (hereafter “tree bats”). Depending on the bat species and habitat type therein, bat response to fire management practices generally is neutral to positive in the southeastern United States [23–26]. Repeatedly burned stands, with reduced overstory clutter and stocking, have increased foraging activity, relative to unburned stands particularly among less maneuverable, larger-bodied bats, or generalist foragers [27–29]. This increased forging activity occurs even though arthropod prey biomass, such as Lepidopterans, may decrease temporarily following burning [30]. However, in reality for bats, the importance of prey availability is often driven by forest structure rather than actual prey abundance. For cavity and exfoliating bark day-roosting species such as those in the genus *Myotis*, burning can both destroy potential roosts [31] and create temporally improved day-roost conditions [32]. However, most research shows fire improves summer day-roosting habitat by increasing the number or relative availability of snags or improving the roosting characteristics of extant live-trees [33–36]. Prescribed fire and improved day-roosting conditions also have been shown to increase the connectedness of bat social networks which is believed beneficial to reproductive success and subsequent juvenile recruitment [37]. Pyrodiversity, or the heterogeneity of post fire conditions, has also been shown to benefit bat communities due to the increased variety of post fire conditions and habitat alterations which meet a greater number of species habitat requirements [38].

Although prescribed fire may improve bat foraging and day-roost habitat conditions in the medium- to long-term, little work has examined the direct effects of fire *in situ* [25]. Though untested, Dickenson et al. [39] cautioned that fires with taller flame heights and intense heat could stress tree-roosting bats due to high carbon monoxide in the smoke plume and/or thermal damage to heat-sensitive tissue such as the patagium and pinna. For dormant season prescribed fires in upland portions of the southeastern United States, i.e., the upper Piedmont and Southern Appalachians physiographic provinces, most hibernating species are presumed to not yet be on the landscape, nor would the migratory tree bats be present in appreciable numbers before the advent of warmer weather [40]. However, this may not be true in the Coastal Plain where current and past legacies of dormant season burning occur when both resident and migratory tree bats are present.

During the dormant season in the southeastern United States, members of the tree bat clade, specifically eastern red bats (*Lasiurus borealis*) and Seminole bats (*Lasiurus seminolus*) day-roost in the foliage of trees, but will occasionally ground-roost within the leaf litter during colder weather where they enter short to medium duration bouts of torpor to reduce energetic demands [41–44]. Anecdotal observations of bats abandoning day-roosts in trees or on the ground ahead of advancing flames during dormant season burning are known [41,45,46]. Regionally, tree-bat ground-roosting in leaf litter occurs when nighttime temperatures approach or fall below freezing and day temperatures fail to exceed 15°C [44,47]. Experimental trials have shown that eastern red bats will arouse from torpor when exposed to smoke or audible fire stimuli, however arousal time was negatively correlated with temperature, suggesting vulnerability or potential mortality from fire during lower ambient temperatures [48–50]. Paradoxically, eastern red bats will raise their metabolism when temperatures approach freezing but do not when ambient temperatures are ≥ 5°C. This means arousal times and risk to fire might be high at low, but still non-freezing, temperatures when managers choose to burn [51]. Although believed to be less impactful than to ground-roosting bats, the consequences of dormant season fire from smoke and flames to bats day-roosting in canopy foliage during warmer temperatures also are largely unknown [25].

The Seminole bat is a North American tree bat that almost exclusively roosts in foliage in pine (*Pinus* spp.) canopies throughout its range in the southeastern United States [43,52,53]. Though widespread during the maternity season, in winter the species’ eastern distribution is concentrated within lower Coastal Plain from South Carolina south throughout the Florida peninsula [54]. Limited data exist on dormant season day-roosting for the Seminole bat, but Hein et al. [43] observed both tree/foliage use as well as ground-roosting during colder weather in coastal South Carolina. Concerns have long existed about the immediate impact to wildlife from prescribed burning, particularly for less-studied taxa and that dormant season burning, though widespread in application, is an ecological “mis-match” with natural fire disturbance processes and regimes in the *s*outheastern United States [22]. With bats facing numerous novel stressors, managers could use better information about the full array of both the positive and potentially negative aspects of dormant season burning in longleaf pine as a stewardship practice. Herein, our objectives were to 1) assess the winter day-roost selection of tree bats in a frequently burned longleaf pine ecosystem; 2) to identify what vegetation characteristics influence the distribution of roosting bats during the dormant season; and 3) to examine how roost selection influences susceptibility to fire effects by assessing the direct response of tree bats to dormant season prescribed fire by describing their behavioral response and subsequent roost selection. We hypothesized that bats would be well adapted to fire and post-burn conditions due to its historic prevalence in the longleaf pine. As such, we expected bats would preferentially roost in locations less infrequently burned and that they would select taller, larger trees that provided optimal solar exposure for the dormant season as well as for reducing the need to evacuate during burning.

## Methods

### Study site

We conducted our study at Camp Blanding Joint Training Center and Wildlife Management Area (CB), Florida, USA, a 227 km^2^ site managed by the Florida Department of Military Affairs and Florida Fish and Wildlife Conservation Commission. Elevations on CB range from 15 m to 74 m asl. Mean annual temperature is 20.5°C and mean annual precipitation is 123.5 cm. Camp Blanding has a subtropical climate characterized by hot humid summers and mild winters. Land use at CB is multi-use for military training, forestry, sand mining, and wildlife management. Major forest types include mesic flatwoods dominated by uneven-aged longleaf pine woodlands, slash pine (*Pinus elliotii*) plantations, xeric sandhills, and riparian bottomland hardwood forests. Proportionally, the portion of CB where our study occurred is 83% pine forests or plantations, 5% upland deciduous forest, 9% bottomland hardwood swamp, 1% upland or wetland shrub, 1% open fields, and <1% developed. In total, 36% of the area is mesic habitat and the remainder is considered xeric. Prescribed burning is used for habitat maintenance or restoration on a three to five-year rotation depending on forest stand composition and installation training needs.

### Field methods

**Fig 1:**
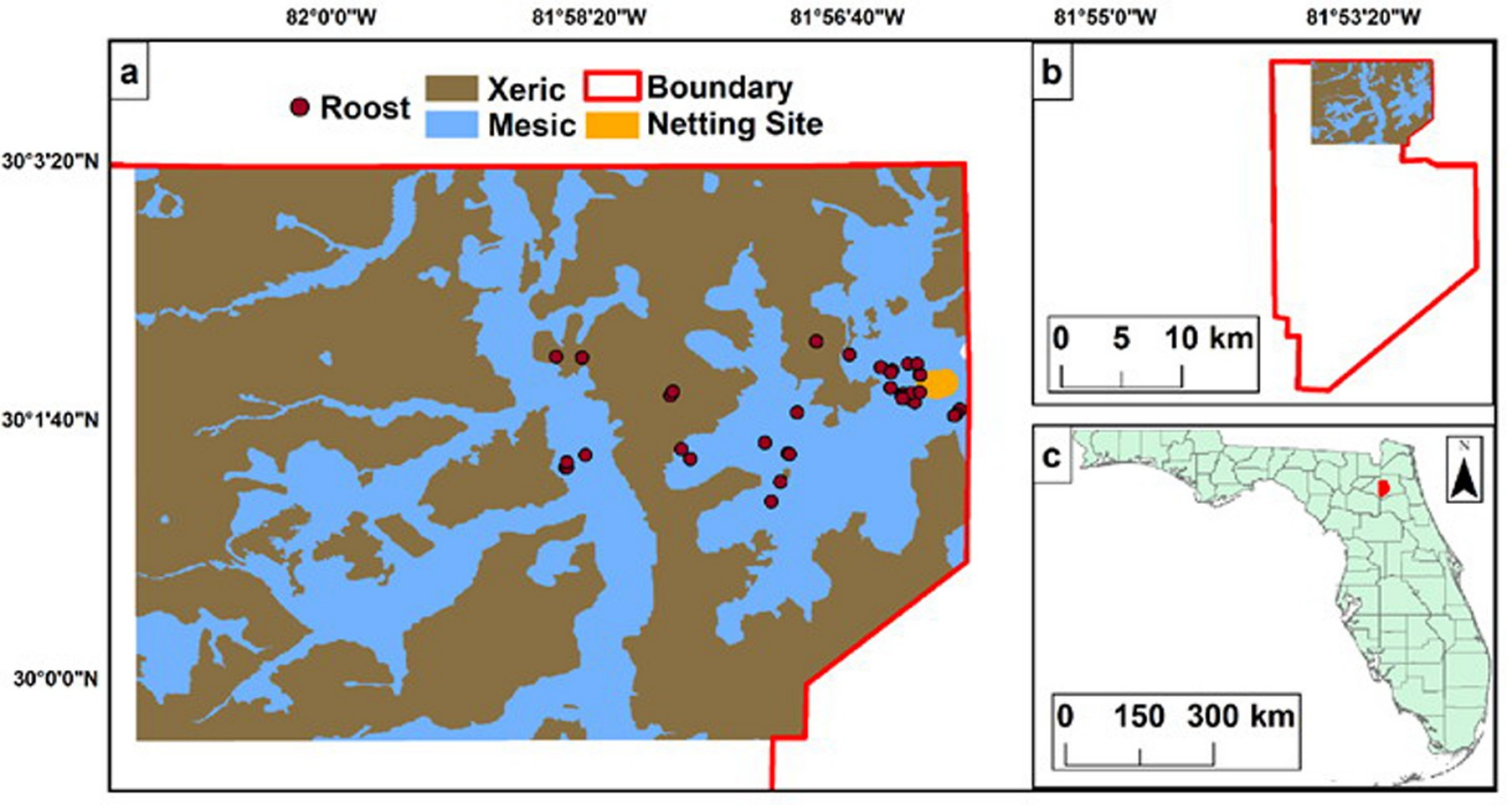
Roost sites (maroon circles) and mist net locations (gold square) relative to mesic (blue) and xeric (brown) forest stands in the area of interest relative to Camp Blanding boundaries (red line), Clay County, Florida from the roost site and tree selection study conducted in February-March 2019 and December-January 2019–2020.

To capture bats, we mist-netted for two nights in February of 2019 and four nights in December of 2019. We erected mist-nets (Avinet, Inc., Dryden, New York) over single-track, unimproved roads, trails, and two retention ponds on CB (Fig 1). Mist-netting was conducted for three to five hours following sunset. For every bat captured, we recorded species, age (by degree of epiphyseal fusion), sex, mass (gm), right forearm length (mm), and reproductive condition [55,56]. We attached uniquely serialized, lipped aluminum bands to the right forearm of all male and the left forearm of all female Lasiurine bats. We affixed a 0.27-gram VHF radio-transmitter (Model LB-2X; Holohil Systems Ltd., Carp, Ontario, Canada) between the scapulae of captured bats using Nu-Hope© (Nu-Hope Laboratories, Inc., Pacoima, California,) or Perma-Type© (The Perma-Type Company, Inc., Plainville, Connecticut) surgical cement. Weight of the transmitter plus glue was < 5 percent of the body mass of radio-tagged bats, as recommended by Aldridge and Brigham [57]. We used TRX-2000 radio telemetry receivers and 3-element Yagi antennas (Wildlife Materials Inc., Murphysboro, Illinois) to locate day-roosts. Tracking was conducted beginning at sunrise from vehicles and then on foot by multiple teams simultaneously and continued for nine days in late February through early March 2019 and then again in early December 2019 until a transmitter dropped from a bat. Once we located a roost, the GPS coordinates were recorded along with a suite of measurements. Following the methods of Silvis et al. [58], for each day-roost located, we recorded roost species, diameter at breast height (DBH), tree height, overstory canopy cover at the day-roost, crown class [59] (i.e., 1 = suppressed, 2 = intermediate, 3 = codominant, 4 = dominant), decay class [60] (i.e.,1 = live, 2 = declining, 3 = recent dead, 4 = loose bark, 5 = no bark, 6 = broken top, 7 = broken bole) and whether roost was in live or dead foliage. We measured the closest four trees, (hereafter, quadrant trees) in each quadrant around the roost tree using the point-quarter method [61]. For each of the quadrant trees, we also recorded species, distance to roost, DBH, tree height, canopy cover, decay class, and crown class. After tracking each morning, roost tree locations were conveyed to the CB natural resource staff who then decided which roost locations were feasible to burn based on current and expected weather conditions, military training schedules, and installation land-management goals. Prescribed burns were then conducted around selected roost trees in the late afternoon. Because holding lines were created near day-roosts with a bulldozer, we monitored bats at day-roosts prior to burning to ensure evacuation was not part of the burning preparation process. Once lines were completed, we used either a strip-head or ring firing techniques depending on the size and shape of the burn unit along with wind direction and speed. During burns, two research team members actively monitored the roost tree(s) using binoculars and telemetry receivers to determine if tagged bats flushed during burns and to record fire behavior. For each burn, we recorded start and end time, bat flush time (when applicable), burn area, and approximations of average flame height, maximum flame height, flame height under the roost, overall smoke production time, and smoke impact time at the roost. If a bat flushed during the burn, we then tracked it to its escape day-roost that afternoon and recorded the same suite of roost measurements described previously. If a bat did not move during a burn, survival was assessed by tracking it to day-roosts on subsequent days.

### Ethics statement

This study was carried out in accordance with state and federal requirements for capture and handling of wildlife. Bat capture and handling protocols were approved by the Animal Care and Use Committee of Virginia Polytechnic Institute and State University (Protocol Number 16–240) and Florida Fish and Wildlife Conservation Commission (Scientific Collecting Permit # LSSC-19-00004). Study sites were located on public land which were accessed by explicit permission of the Florida Department of Military Affairs. Any use of trade, firm, or product names is for descriptive purposes only and does not imply endorsement by the U.S. Government. Data used in this study are archived in the Virginia Polytechnic Institute and State University VTechWorks institutional repository and are available at: https://doi.org/10.7294/JKTV-YV44.

### Fire and environmental variables

To assess the impact of fire and land cover on bat roost area and tree selection [62], we assembled fire history and environmental data from CB historical fire data since 2001 and vegetative cover data to create spatially explicit variables using ArcMap 10.2 (Environmental Systems Research Institute, Inc. Redlands, California). We calculated mean fire return interval by averaging the time between burns in years since 2001, which was therefore a surrogate for the overall fire frequency for any given portion on CB. We created a raster layer contrasting mesic and xeric vegetation by designating forest stands and vegetative communities based on their vegetative alliances (Table 1). We reclassified land cover based on vegetative alliances and dominate tree species into deciduous, pine, swamp, shrub/open and the presence of human structures as urban. Lastly, to understand the real-time effects of prescribed fire on roosting bats we noted the species of bat roosting, whether the bat evacuated or remained during the fire, the average daily temperature during the day of the fire, time to flush since ignition, tree height and tree species.

**Table 1:** Reclassifications of community alliances into mesic or xeric site conditions used as a covariate in a bat day-roost study conducted on Camp Blanding, Clay County, Florida, February-March 2019, and December-January 2019–2020.

Community Alliance	Condition
*Paspalum notatum* Semi-Natural Mowed Grassland Alliance	Xeric
*Pinus elliottii* Planted Forest Alliance	Xeric
*Pinus palustris—(Pinus elliottii)* Forest Alliance	Xeric
*Pinus palustris*—*Pinus (elliottii)* Plantation Xeric Shrubland Phase Alliance	Xeric
*Pinus palustris* / *Quercus spp*. Woodland Alliance	Xeric
*Pinus palustris* Planted Forest Alliance	Xeric
*Pinus palustris* Woodland Alliance	Xeric
*Quercus geminata*—*Quercus myrtifolia—Quercus chapmanii* Shrubland Alliance	Xeric
*Quercus Laevis* Woodland Alliance	Xeric
*Quercus virginiana*—*Pinus palustris—Pinus clausa—Quercus geminata* Forest Alliance	Xeric
*Magnolia virginiana—Nyssa biflora—(Quercus laurifolia)* Saturated Forest Alliance	Mesic
*Magnolia virginiana—Persea palustris* Saturated Forest Alliance	Mesic
*Morella cerifera* Saturated Shrubland Alliance	Mesic
*Nyssa biflora—Acer rubrum—(Liriodendron tulipifera)* Saturated Forest Alliance	Mesic
*Pinus elliottii* Saturated Temperate Woodland Alliance	Mesic
*Pinus palustris—Pinus (elliottii)* Plantation Mesic Shrubland Phase Alliance	Mesic
*Pinus serotina* Saturated Woodland Alliance	Mesic
*Pinus taeda—Liquidambar styraciflua—Acer rubrum* Saturated Forest Alliance	Mesic
*Pinus taeda—Liquidambar styraciflua—Nyssa biflora* Temporarily Flooded Forest Alliance	Mesic
*Quercus* (*phellos*, *nigra*, *laurifolia*) Temporarily Flooded Forest Alliance	Mesic
*Quercus virginiana* Temporarily Flooded Forest Alliance	Mesic
*Spartina bakeri* Seasonally Flooded Herbaceous Alliance	Mesic
*Taxodium ascendens* Seasonally Flooded Forest Alliance	Mesic

### Analyses

To estimate the effects of fire and mesic versus xeric vegetation on roost area selection, we utilized generalized linear models (GLM) in program R [63,64]. We randomly selected 200 points within the northern portion of CB that was within the plausible area where tagged bats might day-roost and contained a wide array of forest and burn conditions using the sample stratified function in the raster package in program R [64,65]. We hypothesized that mean fire return interval, mesic versus xeric vegetation, and land cover would differ between roost points and random points. To assess this, we created seven models with the binomial family and a probit regression link representing all possible combinations of those variables as well as a null model. We fit the models with the GLM function in the stats package in program R and used Akaike Information Criterion (AIC) to compare model weights and determine the top model [66].

To estimate the effects of DBH, tree height, crown class and decay class on roost selection, we utilized generalized linear mixed models (GLMM) with a binomial distribution in package glmmTMB [67] to compare characteristics of roost trees relative to available surrounding trees. Tree height and DBH were highly correlated (R^2^ = 0.79), therefore we opted to use only tree height as the relative measure of tree size. Fixed effects in our model included tree height, class and decay while random effects were bat ID and site ID, this model was then compared to a null model and AIC was used to determine the top model [66]. We also used Because tree species were wholly independent observations relative to each other, we used Pearson’s chi-squared tests [68] to determine if the distribution was equitable among roost tree species to surrounding tree species. To discern the available trees surrounding the roost trees, we used the characteristics of the nearest tree in each of the surrounding quadrants. We assigned significance at alpha = 0.05.

Because there were too few samples to statistically analyze the impact prescribed fire had on day-roost evacuation, we provide qualitative descriptions of the fire conditions, weather conditions, and bat responses. However, if a bat evacuated the roost, we further described the characteristics of the escape roost chosen by the bat. Layne [49] examined response of eastern red bats caught and experimentally placed on a burn site in Missouri and Dickinson et al. [69] recorded responses of two roosting northern long-eared bats (*Myotis septentrionalis*) to prescribed fire. However, to our knowledge, our study was the first of its kind that attempted to document the real-time response of non-hibernating, tree bats in day-roosts of their choosing in a natural setting during prescribed burns. As such, we also provide recommendations for future research attempting to replicate this experiment.

## Results

Over the two sessions, we captured 41 bats [eastern red bat = 1, hoary bat (*Lasiurus cinereus*) = 2, Seminole bat = 13, Southeastern myotis (*Myotis austroripariusi*) = 13, evening bat (*Nycticeius humeralis)* = 3, and tri-colored bat (*Perimyotis subflavus)* = 9]. Of these, we affixed radio-transmitters to 13 Seminole bats, one red bat and two hoary bats. We located 49 Seminole bat roosts thereby allowing for statistical analysis thereof, whereas the low sample sizes of three red bat roosts and four hoary bat roosts from only one individual of each of these species precluded that. These locations accounted for approximately 88% of the possible tagged bat/day combinations during our tracking effort. Dormant-season Seminole bat roosts at CB had a mean DBH of 46.61 cm ± 7.12 SD, tree height of 21.60 m ± 2.68 SD, crown class of 1.7 ± 0.20 SD, and decay class of 1.2 ± 0.21 SD (Table 2). Our model that included tree height, class and decay as well as random effects of bat ID and site ID outcompeted the null model (delta AIC = 54.3). Seminole bats selected dormant season day-roost with greater height, and in higher crown classes than surrounding available trees (Table 2). At the tree scale, Seminole bats day-roosted in loblolly bay (*Gordonia lasianthus*; n = 1), red bay (*Persea borbonia*; n = 7), longleaf pine (n = 15), slash pine (n = 8), and loblolly pine (*Pinus taeda*; n = 12), more than expected based on availability (Table 3). Sweetbay magnolia (*Magnolia virginiana*; n = 1), water oak (*Quercus nigra*; n = 4) and turkey oak (n = 1), were roosted in less than expected based on availability (Table 3). Tree species that were available but never used based on our point-quarter data included bald cypress (*Taxodium distichum*), boxelder (*Acer negundo*), red maple (*Acer rubrum*), American hornbeam (*Carpinus caroliniana*), common persimmon (*Diospyros virginiana*), Carolina holly (*Ilex ambigua*), sweetgum (*Liquidambar styraciflua*), yellow-poplar (*Liriodendron tulipifera*), rusty staggerbush (*Lyonia ferruginea*), Southern magnolia (*Magnolia grandiflora*), black cherry (*Prunus serotina*), swamp white oak.

**Table 2:** Seminole bat (*Lasiurus seminolus*) comparison of dormant season day-roosts with surrounding point-quarter trees (Q TREE) for diameter breast height (DBH) height, crown class, and decay class, and generalized linear mixed model results from Camp Blanding, Clay County, Florida, February-March 2019 and December-January 2019–2020.

		DBH (cm)		Height (m)		Class (1–4)		Decay (1–4)	
Summary		Roost	Q TREE	Roost	Q TREE	Roost	Q TREE	Roost	Q TREE
	Mean	46.61	23.21	21.6	13.28	1.67	2.78	1.22	1.17
	SEM	7.12	1.20	2.68	0.56	0.11	0.20	0.11	0.201
**Results**									
	Estimate	NA		-0.05		-1.06		0.09	
	Std. Error	NA		0.02		0.25		0.09	
	P value	NA		0.01		<0.001		0.34	

Diameter at breast height(DBH) was not included in the model due to correlation with tree height. SEM = Standard mean error. Class (Nyland 1996; i.e., 1 = suppressed, 2 = intermediate, 3 = codominant, 4 = dominant). Decay class (Cline et. Al. 1980; 1 = live, 2 = declining, 3 = recent dead, 4 = loose bark, 5 = no bark, 6 = broken top, 7 = broken bole).

**Table 3:** Chi-square test results by tree species from bat roost site selection study conducted in February-March 2019 and December-January 2019–2020 at Camp Blanding, Clay County, Florida.

	Residuals		Contributions		Use vs. Available
Tree Species	Roost	Quadrant	Roost	Quadrant	
Loblolly bay *Gordonia lasianthus*	1.74	-0.89	5.91	1.55	Greater
Sweetbay magnolia *Magnolia virginiana*	-0.22	0.11	0.1	0.03	Less
Red bay *Persea borbonia*	0.22	-0.11	0.1	0.03	Greater
Slash pine *Pinus elliottii*	1.61	-0.82	5.04	1.32	Greater
Longleaf pine *Pinus palustris*	2.75	-1.41	14.81	3.88	Greater
Loblolly pine *Pinus taeda*	3.31	-1.69	21.36	5.6	Greater
Turkey oak *Quercus laevis*	-1.48	0.76	4.3	1.13	Less
Water oak *Quercus nigra*	-1.65	0.84	5.32	1.39	Less
Other^1^	-3.38	1.73	22.32	5.85	Less
χ^2^	51.17				
*p*-value	<0.001				

^1^Other = bald cypress (*Taxodium distichum*), boxelder (*Acer negundo*), red maple (*Acer rubrum*), American hornbeam (*Carpinus caroliniana*), common persimmon (*Diospyros virginiana*), Carolina holly (*Ilex ambigua*), sweetgum (*Liquidambar styraciflua*), tulip tree (*Liriodendron tulipifera*), rusty staggerbush (*Lyonia ferruginea*), Southern magnolia (*Magnolia grandiflora*), black cherry (*Prunus serotina*), swamp white oak (*Quercus bicolor*), turkey oak (*Quercus cerris*), Shumard oak (*Quercus shumardii*), post oak (*Quercus stellata*), live oak (*Quercus virginiana*), winged elm (*Ulmus alata*), and American elm (*Ulmus americana*).

**Fig 2:**
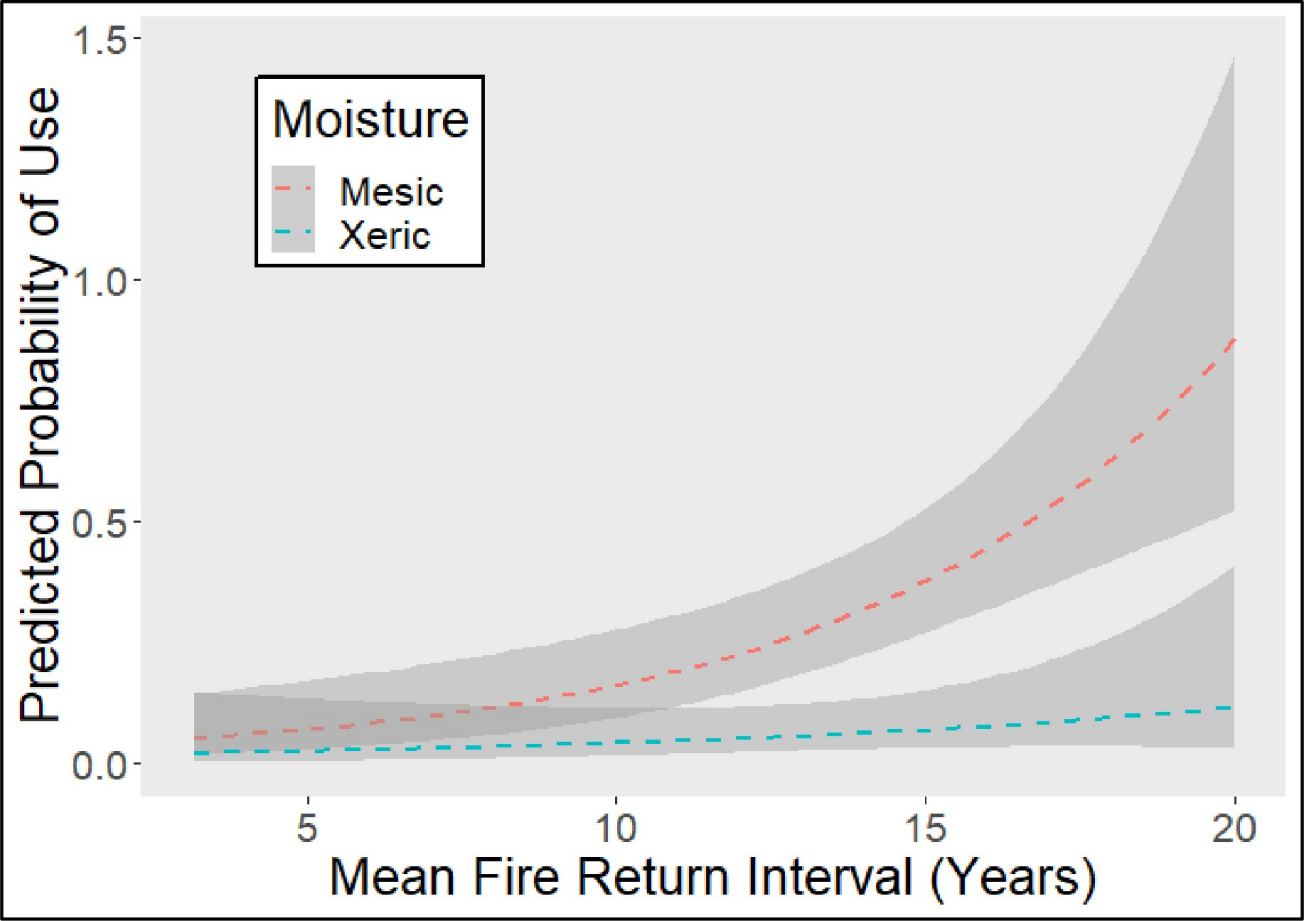
Generalized linear model of predicted probability and 97% confidence intervals of Seminole bat (*Lasiuru seminolus*) day-roost use at the roost area scale from study conducted in February-March 2019 and December-January 2019–2020 on Camp Blanding, Clay County, Florida. Site moisture conditions for habitats as mesic or xeric were designated from forest stands and vegetative communities based on their vegetative alliances.

Residuals with values greater than two denotes a major influence on the chi-square test statistic. Contributions denote the difference between expected and observed values with larger contributions signifying greater difference.

(*Quercus bicolor*), turkey oak (*Quercus cerris*), Shumard oak (*Quercus shumardii*), post oak (*Quercus stellata*), live oak (*Quercus virginiana*), winged elm (*Ulmus alata*), and American elm (*Ulmus americana*). For roost area selection, our top model based on AIC (Table 4) included mean fire return interval and mesic versus xeric vegetation interaction. Seminole bats selected sites with longer mean fire return intervals for day-roosting. There was also a significant interaction between mean fire return interval and mesic versus xeric vegetation in that as fire return interval increased there was a greater disparity between mesic and xeric locations with mesic sites being selected more often (Table 5 and Fig 2). Although there were too few roost sites to analyze for hoary bats, this is the first documentation of hoary bat day-roost sites in Florida during the winter. Of the four hoary bat roost sites we located, one was in loblolly pine whereas the remaining three were in water oak and roosts were located in less fire prone mesic habitat on an alluvial terrace.

**Table 4:** Akaike information criterion table with degrees of freedom for generalized linear models from the Seminole bat (*Lasiurus seminolus*) roost site selection study conducted in February-March 2019 and December-January 2019 on Camp Blanding, Clay County, Florida.

Model	DF	logLik	AIC	Delta	weights
MFRI * Mesic/Xeric	4	-72.71	153.40	0.00	0.88
MFRI + Mesic/Xeric	3	-75.82	157.64	4.23	0.11
Global	7	-74.58	163.15	9.74	0.01
MFRI + Habitat	6	-84.06	180.11	26.70	<0.001
MFRI	2	-89.81	183.61	30.20	<0.001
Mesic/Xeric	2	-93.05	190.10	36.69	<0.001
Mesic/Xeric + Habitat	6	-90.21	192.42	39.01	<0.001
Habitat	5	-110.41	230.81	77.40	<0.001
NULL	1	-123.04	248.08	94.67	<0.001

MFRI = Mean Fire Return Interval in years, Mesic/Xeric = mesic versus xeric vegetative cover, Habitat = land cover reclassified based on vegetative alliances and dominate tree species into deciduous, pine, swamp, shrub/open and the presence of human structures as urban.

**Table 5:** Top generalized linear model of mesic vs xeric habitat and mean fire return interval showing parameter estimates Seminole bat (*Lasiurus seminolus*) day-roost site selection study conducted in February-March 2019 and December-January 2019–2020 at Camp Blanding, Clay County, Florida.

	Estimate	Std. Error	z value	Pr(>|z|)
(Intercept)	-2.46	0.48	-5.05	<0.001
Xeric	0.22	0.62	0.37	0.71
MFRI	0.15	0.02	5.35	<0.001
Xeric:MFRI	-0.10	0.04	-2.51	0.01

Despite our success in locating most day-roosts, the majority of roosts were located in mesic areas that could not be ignited under the weather conditions during either survey session. Nonetheless, we were able to apply six individual prescribed burns that encompassed five Seminole bat, two hoary bat and one eastern red bat day-roosts. From these fires, only one male Seminole bat and one male eastern red bat evacuated their roosts. These two bats flushed approximately ten minutes after the fire was ignited and evacuated only when the fire was near the roost tree, despite smoke reaching the roost prior to that. We observed that both bats flew directly to new day-roosts in adjacent mesic forests, with the Seminole bat moving 147 m and the eastern red bat moving 378 m from the day-roosts within the burns. Both fires that elicited evacuations were the highest intensity of our six burns (max flame heights of 2.43 m and 3.05 m, Table 6). Despite the mean area burned (0.28 ha ± 0.13) per fire being less than what occurs operationally, the smallest area burned (0.09 ha) was one in which a bat evacuated, likely due to a low tree height of 7.3 m (Table 6). As for the bats that did not evacuate during the prescribed burns, their trees had a greater mean height (16.42 m ± 5.03) and were subjected to relatively low fire intensity with lower mean maximum flame heights (1.32 m ± 0.43). Additionally, one Seminole bat roosted within the burn boundaries of the area which had received a prescribed burn the day prior and two other bats (one Seminole and one hoary) switched roosts into areas which were burned the day prior. Another Seminole bat that received two prescribed burns four days apart did not evacuate from either burn despite moving to different roosts at night throughout our study.

**Table 6:** Bat response to roost site prescribed burns with species, burned area, evacuation response, burn start time, time to evacuation, daily temperature average, observed max flame height, tree species and height of tree from burn response study conducted in February-March 2019 and December-January 2019–2020 at Camp Blanding, Florida.

Burn Date	Bat Species	Burn Area (ha)	Evacuated	Time to Evacuation (mins)	Air Temp (C)	Max flame height (m)	Tree Species	Height (m)
02/25/19	LASE^1^	0.44	N	-	18	4	PIPA	19.2
02/25/19	LACI	0.16	N	-	22	3	QUNI	11.8
02/26/19	LASE	0.16	N	-	18	-	PIPA	21
02/26/19	LACI	0.16	N	-	18	-	QUNI	7.6
03/01/19	LASE	0.33	N	-	26	6	PITA	22.5
03/01/19	LASE	0.62	Y	10	27	10	PIEL	13.2
12/11/19	LABO	0.09	Y	10	15	7–8	QUNI	7.32

^1^Slash pine (*Pinus elliottii*) = PIEL, Longleaf pine (*Pinus palustris*) = PIPA, loblolly pine (*Pinus taeda*) = PITA, and water oak (*Quercus nigra*) = QUNI. Red bat (*Lasiurus borealis*) = LABO, hoary bat (*Lasiurus cinereus*), and Seminole bat (*Lasiurus seminolus*) = LASE.

Dashes indicate where maximum flame height estimation was uncertain due to smoke and safety concerns.

## Discussion

Surprisingly, our analysis of Seminole bat day-roosts during the dormant season was congruent with work in the Coastal Plain during the growing season suggesting that minimizing risk from fire and managing thermoregulatory needs are consistent behaviors throughout the year [33,52,70]. For the most part, Seminole bats tended to select larger trees in higher crown classes than surrounding trees, attributable to increased solar exposure to meet thermoregulatory requirements–a factor certainly more critical during the dormant season to maintain homeostasis than during the growing season. We did observe some exceptions with two Seminole bats day-roosting relatively low to the ground in red bay shrubs in open, riparian areas. Unlike Hein et al. [47], our work did not coincide with temperatures low enough to induce bats to use ground-roosts for thermoregulatory benefit.

With the exception of Spanish moss (*Tillandsia usneoides*) use by Seminole bats, our results align with those of Hein et al. [43] whereby bats day-roosted mostly in large pines. Historical records suggest Seminole bats exclusively roost in Spanish moss during winter [71,72], but we found no bats roosting therein, despite being readily available throughout CB. Despite providing insulatory properties during the winter [70], bats in our study likely did not need to use Spanish moss for insulation because minimum temperatures during our study (15°C) never fell as low as Hein et al. [43] observed in coastal South Carolina (-6.8°C to 3.7°C). Our results are more similar to summer study results whereby Seminole bats tended to roost in the terminal branches in the crown of large overstory pine trees [33,52,70]. Because Florida experiences relatively mild winters, it is not surprising that winter roost selection would more closely resemble summer day-roost selection, compared to the greater seasonal differences in roost selection in more northern extents of their range.

All three bat species used day-roosts of fire-adapted tree species, i.e., longleaf pine, slash pine and to a lesser degree, loblolly pine consistent with previous work [33,52,47,70]. Nonetheless, the majority of Seminole bat, and all of the hoary bat day-roosts we found were located in mesic areas with longer fire return intervals rather than in the larger xeric landscape with plantations or natural pine. The importance of these mesic areas and pine-hardwood mixed forests, as well as fire regimes, have been previously noted across a suite of different taxa [73,74], including at CB [75]. Our result finding that bats select more for mesic habitat in areas of less frequent fires supports the hypothesis that roost selection may be a function of seeking to minimize mortality risk from fire caused evacuations. Nevertheless, whether this substantively decreases overall risk is still an open question.

Alternatively, day-roost selection in mesic stands could also be the result of various combined factors such as proximity to water, decreased chance of fire, and increased ability to thermoregulate in colder weather. In other words, roosting in large trees in these mesic locations likely provides a greater range of thermoregulatory options both in terms of solar exposure and abundant mid-story clutter (a result of longer fire-return intervals and lower fire intensity) providing insulation from wind compared to the more open pine savannas in xeric areas, with the added benefit of a lower probability of needing to evacuate during burns which lowers the predator risk associated with moving during the day. Mesic area pines often were close to riparian areas, i.e., bottomland hardwood communities on CB. These areas provide a water resource and foraging habitat for Seminole bats and probably other tree bats in the Coastal Plain [33]. Given our observations, maintaining areas with longer return interval within the longleaf pine ecosystem can create elements of forest structure and composition used by tree bats [33,52,47,70]. Our results provide another example of the benefits of heterogeneity in fire prescriptions for wildlife in longleaf pine systems [76,77] whereby still providing somewhat for fire-adapted species therein [78].

Across three foliage-roosting species we tracked to day-roosts at CB during the dormant season, only two out of seven bats subjected to a prescribed fire evacuated their roosts during prescribed burns. In both cases, the roost conditions might have influenced the need to evacuate, as the bats were in relatively lower roosts than we observed for other tagged bats and were exposed to higher fire intensities. Interestingly, when these two individuals evacuated they did so to mesic locations despite flying over suitable day-roost conditions in surrounding unburned xeric habitat–perhaps indicating an ability to assess the vulnerability of the site to continue to carry the fire. Despite dormant season burning being outside the natural historical norm, it did occasionally occur and this may have allowed bats to adapt to fire across both growing and dormant seasons. As such, an adaptation to find a low fire risk site makes evolutionary sense regardless of season. Bats that roosted in more fire-prone areas risk expending energy for evacuation or are more likely to be depredated during diurnal movement [79,80]. As for the five bats that did not evacuate during the prescribed burns, all roosted in taller trees and experienced less intense fire effects than surrounding areas lower in the canopy. Besides heat and fire, smoke has also been suggested to be detrimental to bats [39,69], but we observed that smoke quickly dissipated and it may have been cooler than growing season smoke. Furthermore, smoke has been suggested to be an indicator of fire threat [81] and in our study may have indicated low threat due to poor combustion and cooler smoke.

Additionally, some bats switched roosts into areas that were burned the day prior, and one bat never evacuated even after experiencing multiple burns. We suggest that fire not only has minimal negative consequences during a burn in this setting, but that the short-term post-fire conditions created may be also be beneficial for roosting bats [37,82]. Smoldering embers as well as the decreased albedo, the percent of solar radiation reflected at the soil surface, generate higher temperatures than unburned areas [83] which could benefit bat thermoregulation during the winter. Furthermore, these sites provide roost areas with minimal risk of fire. The use of fire as a habitat management tool is believed to positively impact a wide range of bat species in the southeastern United States [25,27,29,30]. Outside of the southeastern United States, bat response often has focused on the wildfire rather than prescribed fire impacts on foraging activity, bat community structure and loss of habitat [38,84,85]. However, in Australia, the lesser long-eared bat (*Nyctophilus geofffroyi*) were shown to have shorter torpor bouts and longer normothermia duration 4 months post-fire compared to 2 years post- fire, allowing them to save energetic resources more in the 4 month post fire period [86]. The blackening of the landscape can increase roost temperature due to solar radiation thereby requiring less energetic output to maintain body temperatures [87]. Given our results, we posit that the probability of evacuation is likely driven by the interaction of roost height and fire intensity with an increasing probability of evacuation with increasing fire intensity and decreasing roost height, which may be ultimately driven by the amount of heat reaching the roost. Despite previous studies recording bats roosting in the leaf litter [41,43,47], we found no bats roosting in the leaf litter, which may have been due to temperatures not reaching the low threshold that triggers leaf litter roosting.

The complex nature of this study required scientific research protocols, prescribed fire regulations and requirements, as well as optimal weather conditions to all be attained for data collection to occur. As such, many of our efforts were focused on proof of concept for the methods to expand this type of research more broadly; thus, resulting in meaningful, but coarse, data collection. With our observations, future studies attempting to replicate this study should attempt to increase sample sizes of radio-tagged bats particularly across areas more suitable to operational burning, and to more accurately measure fire and weather conditions and assess the effects across a latitudinal gradient to understand differences across temperatures and ecoregions. For example, moving farther north, where bats are more likely to roost in leaf litter due to colder temperatures, could provide more opportunities for this type of research and allow for a better understanding of the evacuation probabilities and mortality risks associated with leaf litter roosting when subjected to burning [43,47,49]. Furthermore, conducting experimentation in less fire-prone ecosystems, but where fire is still applied, such as the Piedmont physiographic portion of the southeastern United States or where fire intensity can be greater such in montane ponderosa pine (*Pinus ponderosa*) in the southwestern United States, as well as other temperate to warm-temperate fire-adapted forest types elsewhere would increase our understanding of bat adaptation to fire across the spectrum of current fire regimes and ecosystems. Nevertheless, our data provide a valuable case study that begins to elucidate the function of vegetation attributes selected by tree bats for roost sites and highlights the importance of minimizing fire susceptibility in this selection process.

## Conclusion

Bats in fire-dominated landscapes have evolved with fire and our results suggest minimal negative responses to dormant season fire. As such, we suggest the continued use of fire during the dormant season to benefit broader habitat restoration goals and long-term positive effects of bats. Because the majority of roosts occurred in pine trees that are fire-adapted and maintained on the landscape in part by fire, we suggest a consistent but infrequent prescription of fire be maintained in mesic locations to promote some pine regeneration while maintaining the longer fire intervals selected for by Seminole bats and perhaps other tree bats. Nonetheless, when fire intensity is great or when tree height is low (young-aged or mid-rotation stands), evacuation may occur. Collectively, our results may suggest a behavioral mechanism for roosting bats to mitigate the negative responses to fire in a fire-prone landscape by generally roosting in mesic areas in this part of the Coastal Plain. However, when the sites do burn, as in our study, roosts were situated high enough to not be affected by fire. When that was not the case as we observed in two xeric sites, bats evacuated to taller roosts in mesic areas.
